# NOBLE and EXCEL: The debate for excellence in dealing with left main stenosis

**DOI:** 10.21542/gcsp.2018.3

**Published:** 2018-03-14

**Authors:** Hamood Al Kindi, Amir Samaan, Hatem Hosny

**Affiliations:** 1 Aswan Heart Centre, Aswan, Egypt; 2 Sultan Qaboos University Hospital, Muscat, Sultanate of Oman; 3 Cairo University, Cairo, Egypt

## Abstract

Left main coronary artery (LMCA) disease is associated with increased morbidity and mortality. Coronary artery bypass grafting surgery (CABG) has always been the standard revascularization strategy for this group of patients. However, with the recent developments in stents design and medical therapy over the past decade, several trials have been designed to evaluate the safety and efficacy of percutaneous coronary intervention (PCI) as an alternative to CABG surgery in patients with LMCA disease. Recently, the results of two major trials, EXCEL and NOBLE, comparing CABG versus PCI in this patient population have been released. In fact, the results of both trials might appear contradictory at first glance. While the EXCEL trial showed that PCI was non-inferior to CABG surgery, the NOBLE trial suggested that CABG surgery is a better option. In the following review, we will discuss some of the similarities and contrasts between these two trials and conclude with lessons to be learned to our daily practice.

## Introduction

Left main coronary artery (LMCA) disease is a life-shortening disease with significant morbidity and mortality. LMCA stenosis present in about 15% of patients with symptomatic ischemic *heart disease*^1^. Surgical revascularization with coronary artery bypass grafting (CABG) remain the gold slandered treatment in left main and triple vessels disease. However, after the introduction of percutaneous coronary intervention (PCI) in 1977, there was a growing interest in treating left main disease using percutaneous approach with the aim of decreasing the morbidity associated with CABG.

Both the European^2^ and American^3^ guidelines strongly recommend (Class I) CABG as the treatment of choice for unprotected left main disease (UPLMCA), while the recommendation for PCI depends on the anatomical complexity of the coronary artery disease, based on the SYNTAX score and the surgical risk of the patients. These recommendations were based mainly on the results of the LM subgroup analysis of SYNTAX trial (705 patients) that showed no difference in the overall major adverse cardiac and cerebrovascular events between CABG and PCI in patients with LM disease.

PCI-treated patients had a lower stroke but a higher revascularization rate versus CABG. However, this was a subgroup analysis^4^ from the original trial that compared CABG and PCI with first-generation paclitaxel-eluting stents (PES) in the treatment of triple vessel and LM disease. This trial included both randomized and non-randomized patients and its primary results showed that PCI with was inferior to CABG for the endpoint of major adverse cardiac and cerebrovascular events (MACCE: death, MI, stroke, or unplanned repeat revascularization)^5^.

In 2015, the 5-year outcomes of the PRECOMBAT trial (600 patients) were published, comparing PCI to CABG in the treatment of unprotected LM disease. The two groups did not differ significantly in terms of death, myocardial infarction or stroke. Ischemia-driven revascularization occurred more frequently in the PCI group than in the CABG group^6^.

Furthermore, LE MANS trial (105 patients) compared PCI and CABG in patients with unprotected left main disease with low or medium syntax score. In this trial, 35% of the patients in the PCI group receive drug-eluting stents whereas arterial grafts to the left anterior descending artery were utilized in 81% of the patients. The primary end point of the study was left ventricular ejection fraction (LVEF) that was slightly higher in the PCI group compared to CABG group, but not statistically significant (55% vs. 50%, *p* = 0.07). The rate of MACCE was also not significant between the two groups^7^.

These trials were not adequately powered to study the difference between the two treatment modalities for left main coronary artery disease. Furthermore, the introduction of the new generation drug eluting stents with proven safety and efficacy prompted the design of two large randomized trials: The Nordic-Baltic-British left main revascularization study (NOBEL) and Everolimus-Eluting Stents or Bypass Surgery for Left Main Coronary Artery Disease (EXCEL), that published their results in December 2016 in *The Lancet*^8^ and *New England Journal of Medicine*^9^, respectively.

## NOBLE

### Design

The Nordic-Baltic-British left main revascularization study (NOBLE) is a prospective, randomized, open-label, non-inferiority trial, done at 36 centers in Europe. Patients were randomized to receive either CABG or PCI. This trial includes patients with stable angina, unstable angina or non-ST elevation myocardial infraction (NSTEMI).

LM lesions were visually assessed with diameter ≥50% or fractional flow reserve ≤0.80 in different segments of the left main coronary artery. SYNTAX score was calculated and all patients with low, medium and high score were included. Patients were treated with the intention of achieving complete re-vascularization of all vessels with significant lesions. Biolimus-eluting stent was the recommended stent in this trial. This stent is biodegradable and useful for large diameter vessel.

In the PCI group, ostial and mid-shaft lesions were treated with a single stent. Distal bifurcation lesions could be treated with various techniques preferably by the ‘culotte’ technique. Intravascular ultrasound was strongly recommended pre- and post-stent deployment, but was not mandatory.

In the CABG group, the left internal mammary artery was recommended for re-vascularization of the left anterior descending coronary artery and for the other lesions, saphenous venous grafts, free arterial grafts, or the right internal mammary artery could be used. Treatment included 75–150 mg of aspirin, lifelong. In both groups, patients with acute coronary syndrome received 75mg clopidogrel daily for 12 months. All patients in the PCI groups also received 75mg clopidogrel daily for 12 months.

The primary endpoint was a composite of MACCE (death from any cause, non-procedural myocardial infarction, repeat revascularization, or stroke). The intention-to-treat principle was used in the analysis used in this trial.

### Results

1184 patients were included in the analysis (592 patients in each group). Patients were followed for at least 1 year and extended follow-up was available for a median of 3.1 years. The mean age was 66.2 yrs in both groups. The logistic EUROSCORE was 2 in both groups and the SYNTAX scores were similar between the two groups (22.4 in the PCI group and 22.3 in the CABG group). About 80% of the patients in each group had stable angina or silent ischemia. Distal left main coronary artery disease was a common presentation (81%) in both groups. CABG was performed with the on-pump technique in 84% of patients, and 96% of patients underwent arterial grafting of the left anterior descending artery.

Kaplan–Meier estimates of MACCE were significantly higher in PCI (28%) compared to CABG (18%). The rate of myocardial infarction and revascularization (mainly de novo revascularization) was significantly higher in PCI group compared to CABG, but the overall mortality and stroke were not statistically significant.

At 30 days, the stroke rate in PCI group was significantly less than in the CABG group but this difference was not seen at 1- and 5-year follow-up. Disadvantages of CABG manifested during the first 30 days due higher blood transfusion rate, reoperation for bleeding and reoperation for sternum infection.

Contrary to previous studies, the SYNTAX score was not associated with adverse outcomes after PCI compared to CABG. Figure 2 illustrates the difference in outcomes according to the SYNTAX score between the NOBLE trial and SYNTAX trial. This may indicate that SYNTAX score is useful for patients with triple vessel disease rather than isolated LM disease. In conclusion, the NOBLE trial showed that CABG might provide a better clinical outcome for treatment of left main coronary artery disease than PCI.

## EXCEL

### Design

The EXCEL trial was a prospective randomized open-label, non-inferiority trial undertaken at 126 centers in 17 countries around the world. Patients were randomized to receive either CABG or PCI. Patients who had stable and unstable angina were included in the study, however patient who were having myocardial infarction were excluded. Patients were included if they had left main stenosis of 70% assessed visually, or 50%-70% determined by means of invasive or non-invasive methods. In addition, patients with left main equivalent were included in the trial.

SYNTAX score was determined and patients who had score of higher than 33 were excluded. Complete revascularization was the intention of treatment in both groups. A second-generation drug eluting stent (fluoropolymer-based cobalt–chromium everolimus eluting stent) was used in this study. The trial included randomized patients and patients’ registry for those who didn’t meet the inclusion criteria.

Distal bifurcating lesions were treated with a two-stent strategy using various techniques. CABG was performed both on and off pump, with the aim of complete revascularization for vessels with 50% stenosis. Arterial grafts were strongly recommended. Patients in the PCI group received dual antiplatelet therapy for 1 year. In the CABG group, aspirin was administered during the perioperative period and the clopidogrel was used during follow up, but was not mandatory.

The primary end point was a composite of death from any cause, stroke, or myocardial infarction at 3 years. Secondary objectives were the rate of a composite of death from any cause, stroke, or myocardial infarction at 30 days and the rate of a composite of death, stroke, myocardial infarction, or ischemia-driven revascularization at 3 years. The intention-to-treat principle was used in the analysis used in this trial.

### Results

1905 patients underwent randomization, 948 were assigned to the PCI group and 957 to the CABG group. Baseline clinical and angiographic characteristics were similar between the groups. The SYNTAX score according to assessment at local sites was low (≤22) in 60.5% of the patients and intermediate (23–32) in 39.5% of the patients. Distal left main lesion was present in 80.5% of the patients. Double or triple vessel coronary artery disease was present in 51.3% of the patients. Intravascular ultrasonographic imaging guidance was used in nearly 80% of the patients in the PCI group. Medications at 3 years were different between the two groups. The use of dual antiplatelets was significantly higher in the PCI group compared to CABG group. The median duration of follow-up was 3 years in both groups. There was no difference between the two groups in respect to the primary composite end-point event of death, stroke, or myocardial infarction at 3 years (15.4% of the patients in the PCI group and in 14.7% of the patients in the CABG group).

At 30 days, the composite end-point event of death, stroke, or myocardial infarction had occurred less in the PCI group compared to the CABG group (4.9 vs 7.9, *p* = 0.008). Major and minor bleeding events were also less common after PCI than after CABG. At 3 years, the composite endpoint event of death, stroke, myocardial infarction, or ischemia-driven revascularization had occurred in 23.1% of the patients in the PCI group and in 19.1% of the patients in the CABG group.

Ischemia-driven revascularization during follow-up was more frequent after PCI than after CABG (in 12.6% vs. 7.5% of the patients, *P* < 0.001). The difference was more distinct in the non-target lesion revascularization were in the CABG the rate was 1.3% compared to 5.7% in the PCI group. Notably, stent thrombosis occurred in only 0.7% of patients within 3 years after the procedure and was less common than symptomatic graft occlusion.

In summary, the results of EXCEL trial suggest that PCI with everolimus-eluting stents is an acceptable, or perhaps preferred, alternative to CABG in patients with left main coronary artery disease with low or intermediate SYNTAX score.

## Discussion

**Figure 1.**
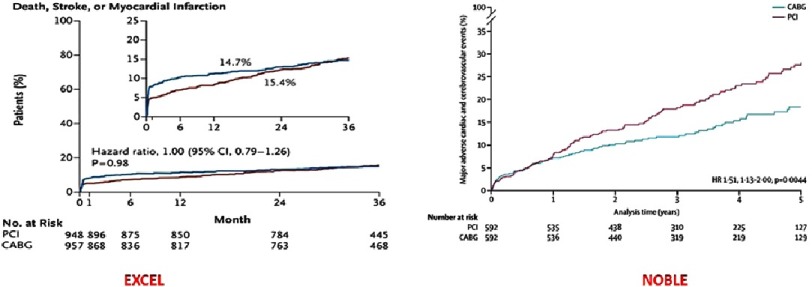
Primary end points of EXCEL and NOBLE trials^8,9^.

Left main coronary artery disease is associated with high morbidity and mortality owing to the large territory at risk for myocardial ischemia. Coronary artery bypass surgery remains the gold standard treatment option for patients with significant left main disease or triple vessel disease. Since the introduction of PCI, there has been numerous studies that compared the two treatment options that address the same question with almost similar and expected results^10,11^. The comparison between CABG and PCI will remain a moving target because of the continuous development of stent design with the aim of improving the patency and decreasing the incidence of stent thrombosis. This new development prompted the design the two dedicated randomized trials comparing CABG and PCI in LM disease: NOBEL and EXCEL. Table 1 and Figure 1 summarizes the key features and results of these two trials.

**Table 1:** Comparison of EXCEL and NOBLE trials.

	EXCEL	NOBLE
**Inclusion criteria**	- Significant unprotected left main coronary artery (ULMCA) disease or left main equivalent disease - Clinical and anatomic eligibility for both PCI and CABG as agreed to by the local Heart Team - Silent ischemia, stable angina, unstable angina, recent MI with normalization of CK-MB prior randomization - In addition to randomized patients it also include universal registry.	- Stable, unstable angina pectoris or Acute coronary syndrome - Significant unprotected left main coronary artery (ULMCA) with no more than three additional non-complex PCI lesions - Patient eligible to be treated by CABG and by PCI
**Main exclusion criteria**	- Prior PCI of the left main at any time prior to randomization or prior PCI of any other (non-left main) coronary artery lesions within one year prior to randomization - Prior CABG - Need for any concomitant cardiac surgery - Inability to receive dual antiplatelet therapy for at least one year - Pregnancy or intention to become pregnant - Life expectancy less than 3 years	- ST-elevation infarction within 24 h - Patient is too high risk for CABG - Expected survival less than one year - Allergy to aspirin, clopidogrel or ticlopidine
**Angiographic exclusion criteria**	- SYNTAX score ≥33 - Visually estimated left main reference vessel diameter < 2.25 mm or > 4.25 mm (post-dilatation up to 4.5 mm is allowed)	- CABG clearly better treatment option (LMCA stenosis and >3, or complex additional coronary lesions)
**Primary end point**	- Death, MI and stroke	- Death, stroke, non-procedural MI and new revascularisation (PCI or CABG)
**Sample size**	1,905 patients	1,200 patients
**Participating centres**	131 active sites worldwide	36
**Main results**	At 3 years, a primary end-point event had occurred in 15.4% of the patients in the PCI group and in 14.7% of the patients in the CABG group	At 5 years, primary end points occurred in 28% of the patients in PCI group and in 18% of the patients in the CABG group
**Conclusion**	In patients with left main coronary artery disease and low or intermediate SYNTAX scores, PCI was non inferior to CABG	CABG might be better than PCI for treatment of left main stem coronary artery disease.

NOBEL and EXCEL trials are both non-inferiority randomized trials that compare PCI and CABG in the treatment of left main disease. The EXCEL trial included also 1000 patients as a registry for those who cannot be randomized to either PCI or CABG. The EXCEL trial was larger in number (1905 patients from 131 centers around the world) compared to 1200 patients from 36 centers in the NOBLE trail. Patients with left main stenosis with low to high syntax score were included in NOBLE, however in the EXCEL patients with high SYNTAX score were excluded. The stent used in the two trials were different. Biolimus-eluting biodegradable stent was used in the NOBLE trial while fluoropolymer-based cobalt–chromium everolimus eluting stent was used in the EXCEL trial. The primary end points were different between the two trials. MACCE (death, MI, stroke and repeat revascularizations) were the primary end points in the NOBLE trial, while in the EXCEL trial repeat revascularization was not part of the primary end points. The two trials gave two contradicting conclusions. The NOBLE trial showed that CABG is superior to PCI in the treatment of left main disease. The EXCEL trial showed that PCI is a valid alternative option and comparable to CABG in selected patient of left main disease.

**Figure 2.**
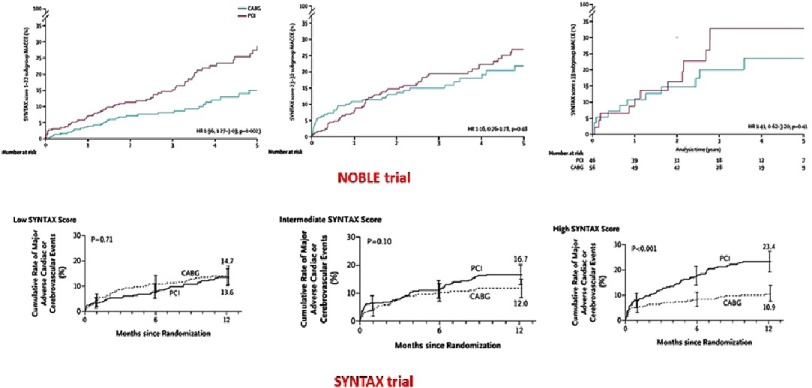
Outcomes according to the SYNTAX score in NOBLE and SYNTAX trials^8,16^.

## What we have learned?

The results of the NOBLE and EXCEL trials give cardiac surgeons and cardiologists the best evidence available in the treatment of LMCA stenosis. The low mortality and morbidity in these two trails demonstrate the continuous improvement of both the stent industry (with lower rates of stent thrombosis) as well refinements of surgical techniques for coronary revascularization. The rate of revascularization, especially de novo lesion revascularization, continue to be lower in CABG compared to PCI, similar to what has been shown in previous trials. The consequences of repeat revascularization might be detrimental. It has been demonstrated that repeat revascularization to be an independent predictor for late outcome of death, stroke and myocardial infarction based on the 5 years results of the SYNTAX trial^12^.

The NOBLE trial provides a direct comparison between CABG and PCI in the treatment of left main disease with different complexities, while the hypothesis of EXCEL trial is closer to current practice, which includes patients with low to intermediate syntax score. Lee et al. compared the results of the EXCEL and NOBLE trials with Left MAIN Revascularization (IRIS–MAIN) registry which resemble real life setting. They found that the EXCEL trial might represent better generalizability with respect to baseline characteristics and observed clinical outcomes compared with the NOBLE trial^13^.

The debate between CABG and PCI will remain unchanged and will be influenced by constant development within the stent industry. Clinical practice will be influenced by our interpretation of current evidence and guidelines, along with the demand of the patients to have the least invasive procedures^14^. It has been demonstrated from a cardiac catheterization database, that patients with coronary artery disease receive more recommendations for PCI and fewer recommendations for CABG surgery than indicated in the guidelines^15^.

Finally, the heart team approach in treating left main coronary artery disease is important and will result in better strategies to have better informed consent by having honest discussions with patient about the advantages and the disadvantages of each treatment option. We should recognize that every study or trial has its own bias and we should make sure that patients are not included in that bias by the practicing a personalized clinical approach.
